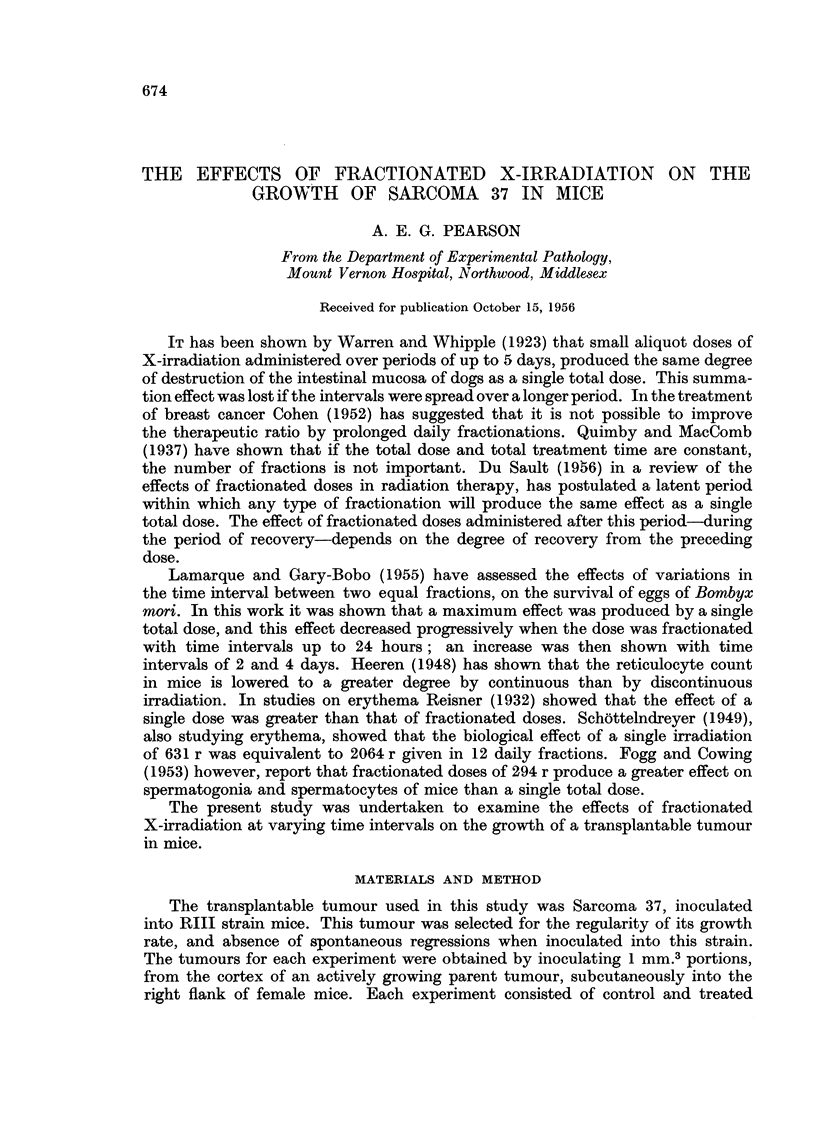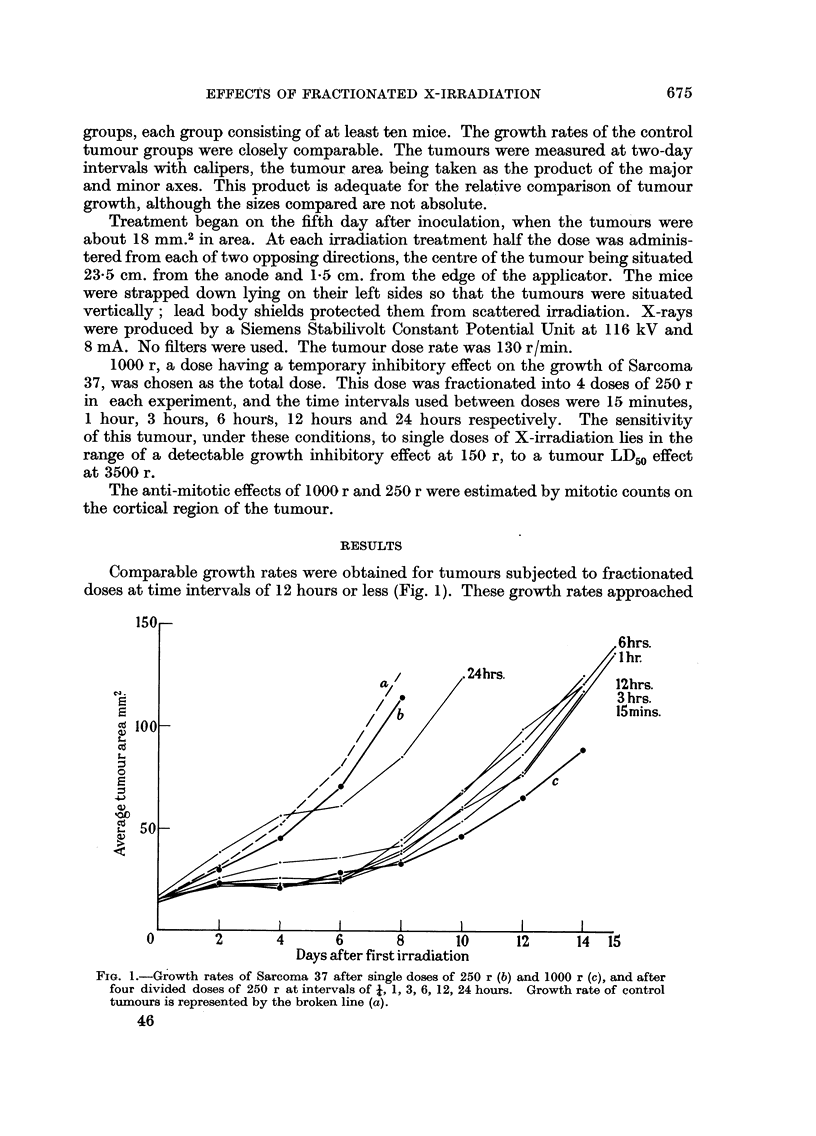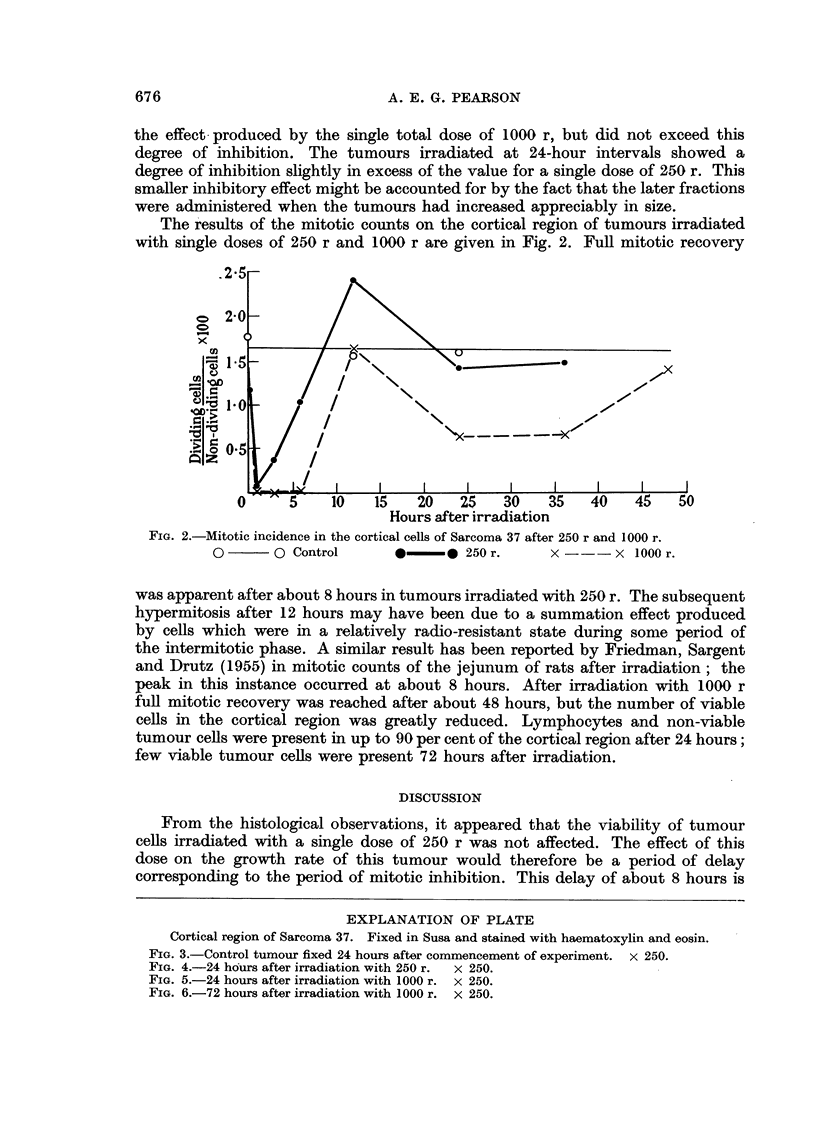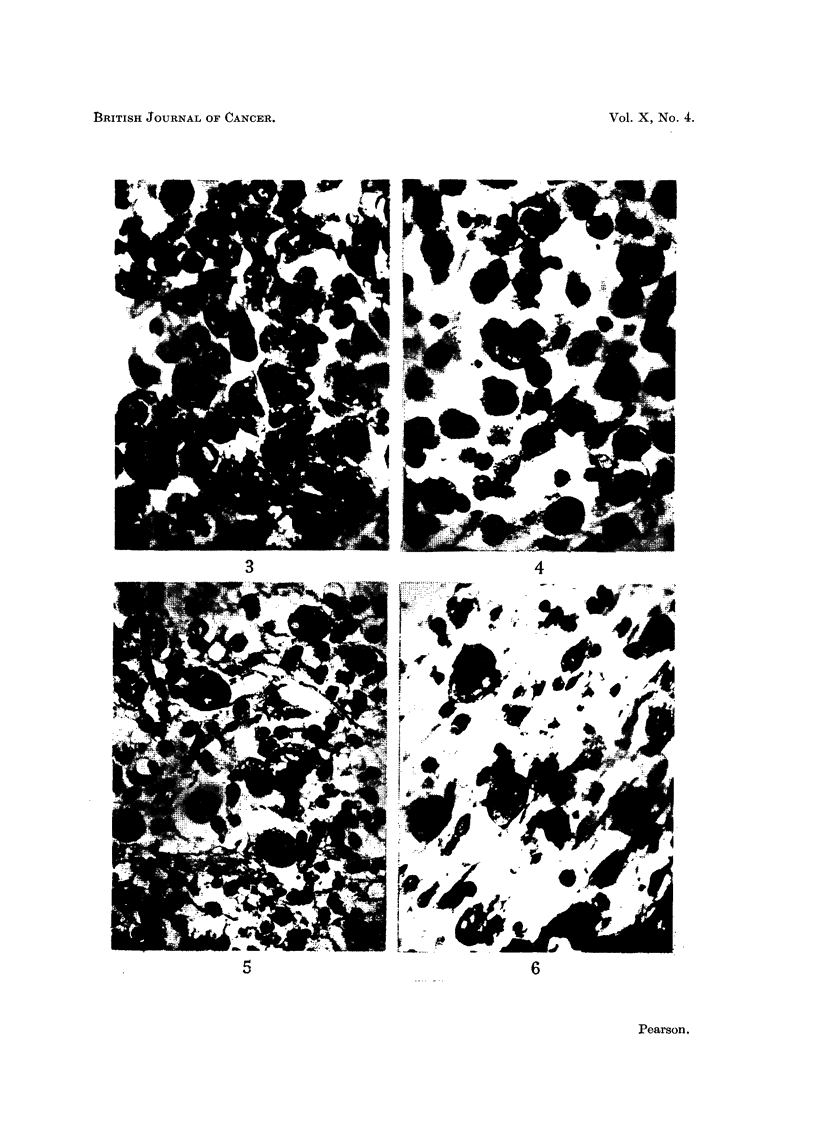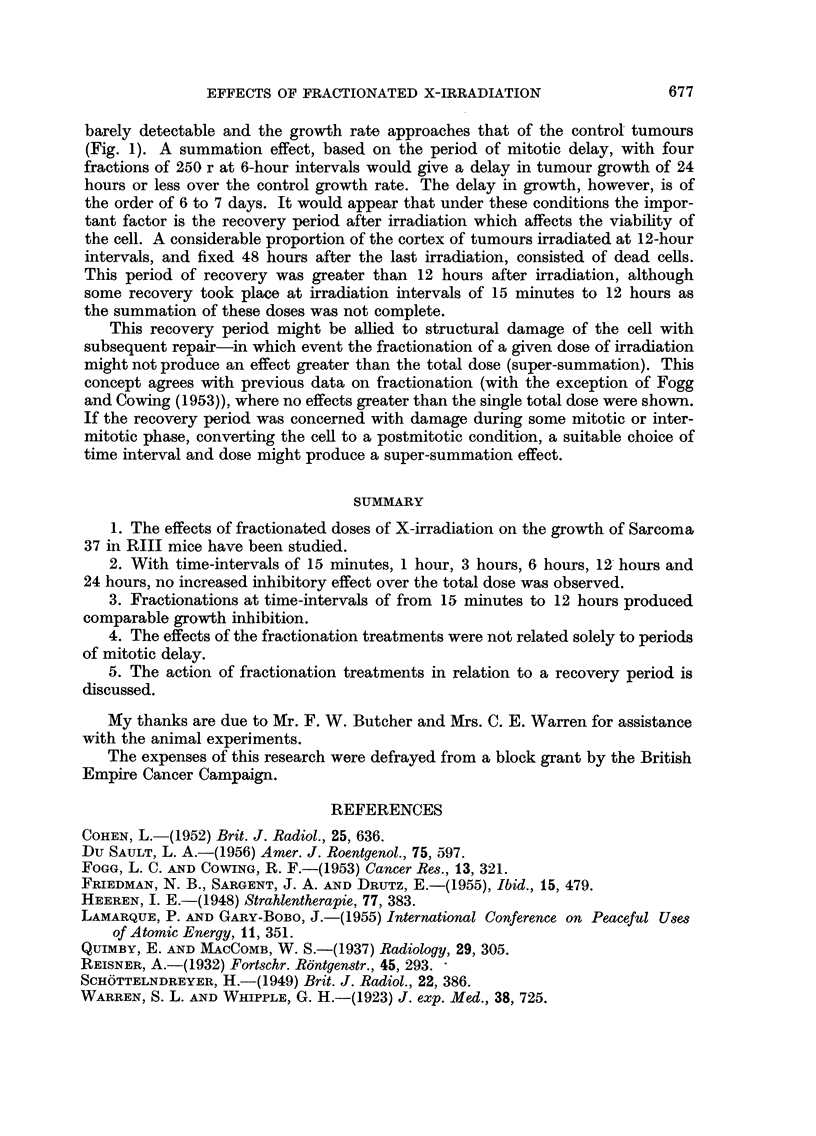# The Effects of Fractionated X-Irradiation on the Growth of Sarcoma 37 in Mice

**DOI:** 10.1038/bjc.1956.81

**Published:** 1956-12

**Authors:** A. E. G. Pearson

## Abstract

**Images:**


### THE EFFECTS OF FRACTIONATED X-IRRADIATION ON THE GROWTH OF SARCOMA 37 IN MICE

A. E. G. PEARSON

*From the Department of Experimental Pathology, Mount Vernon Hospital, Northwood, Middlesex*

Received for publication October 15, 1956

IT has been shown by Warren and Whipple (1923) that small aliquot doses of X-irradiation administered over periods of up to 5 days, produced the same degree of destruction of the intestinal mucosa of dogs as a single total dose. This summation effect was lost if the intervals were spread over a longer period. In the treatment of breast cancer Cohen (1952) has suggested that it is not possible to improve the therapeutic ratio by prolonged daily fractionations. Quimby and MacComb (1937) have shown that if the total dose and total treatment time are constant, the number of fractions is not important. Du Sault (1956) in a review of the effects of fractionated doses in radiation therapy, has postulated a latent period within which any type of fractionation will produce the same effect as a single total dose. The effect of fractionated doses administered after this period—during the period of recovery—depends on the degree of recovery from the preceding dose.

Lamarque and Gary-Bobo (1955) have assessed the effects of variations in the time interval between two equal fractions, on the survival of eggs of *Bombyx mori*. In this work it was shown that a maximum effect was produced by a single total dose, and this effect decreased progressively when the dose was fractionated with time intervals up to 24 hours ; an increase was then shown with time intervals of 2 and 4 days. Heeren (1948) has shown that the reticulocyte count in mice is lowered to a greater degree by continuous than by discontinuous irradiation. In studies on erythema Reisner (1932) showed that the effect of a single dose was greater than that of fractionated doses. Schöttelndreyer (1949), also studying erythema, showed that the biological effect of a single irradiation of 631 r was equivalent to 2064 r given in 12 daily fractions. Fogg and Cowing (1953) however, report that fractionated doses of 294 r produce a greater effect on spermatogonia and spermatocytes of mice than a single total dose.

The present study was undertaken to examine the effects of fractionated X-irradiation at varying time intervals on the growth of a transplantable tumour in mice.

### MATERIALS AND METHOD

The transplantable tumour used in this study was Sarcoma 37, inoculated into RIII strain mice. This tumour was selected for the regularity of its growth rate, and absence of spontaneous regressions when inoculated into this strain. The tumours for each experiment were obtained by inoculating 1 $mm.^3$ portions, from the cortex of an actively growing parent tumour, subcutaneously into the right flank of female mice. Each experiment consisted of control and treated groups, each group consisting of at least ten mice. The growth rates of the control tumour groups were closely comparable. The tumours were measured at two-day intervals with calipers, the tumour area being taken as the product of the major and minor axes. This product is adequate for the relative comparison of tumour growth, although the sizes compared are not absolute.

Treatment began on the fifth day after inoculation, when the tumours were about 18 mm.$^2$ in area. At each irradiation treatment half the dose was administered from each of two opposing directions, the centre of the tumour being situated 23·5 cm. from the anode and 1·5 cm. from the edge of the applicator. The mice were strapped down lying on their left sides so that the tumours were situated vertically ; lead body shields protected them from scattered irradiation. X-rays were produced by a Siemens Stabilivolt Constant Potential Unit at 116 kV and 8 mA. No filters were used. The tumour dose rate was 130 r/min.

1000 r, a dose having a temporary inhibitory effect on the growth of Sarcoma 37, was chosen as the total dose. This dose was fractionated into 4 doses of 250 r in each experiment, and the time intervals used between doses were 15 minutes, 1 hour, 3 hours, 6 hours, 12 hours and 24 hours respectively. The sensitivity of this tumour, under these conditions, to single doses of X-irradiation lies in the range of a detectable growth inhibitory effect at 150 r, to a tumour $LD_{50}$ effect at 3500 r.

The anti-mitotic effects of 1000 r and 250 r were estimated by mitotic counts on the cortical region of the tumour.

### RESULTS

Comparable growth rates were obtained for tumours subjected to fractionated doses at time intervals of 12 hours or less (Fig. 1). These growth rates approached

Fig. 1.—Growth rates of Sarcoma 37 after single doses of 250 r (*b*) and 1000 r (*c*), and after four divided doses of 250 r at intervals of ¼, 1, 3, 6, 12, 24 hours. Growth rate of control tumours is represented by the broken line (*a*).

the effect produced by the single total dose of 1000 r, but did not exceed this degree of inhibition. The tumours irradiated at 24-hour intervals showed a degree of inhibition slightly in excess of the value for a single dose of 250 r. This smaller inhibitory effect might be accounted for by the fact that the later fractions were administered when the tumours had increased appreciably in size.

The results of the mitotic counts on the cortical region of tumours irradiated with single doses of 250 r and 1000 r are given in Fig. 2. Full mitotic recovery

FIG. 2.—Mitotic incidence in the cortical cells of Sarcoma 37 after 250 r and 1000 r.
○——○ Control ●——● 250 r. ×— — —× 1000 r.

was apparent after about 8 hours in tumours irradiated with 250 r. The subsequent hypermitosis after 12 hours may have been due to a summation effect produced by cells which were in a relatively radio-resistant state during some period of the intermitotic phase. A similar result has been reported by Friedman, Sargent and Drutz (1955) in mitotic counts of the jejunum of rats after irradiation ; the peak in this instance occurred at about 8 hours. After irradiation with 1000 r full mitotic recovery was reached after about 48 hours, but the number of viable cells in the cortical region was greatly reduced. Lymphocytes and non-viable tumour cells were present in up to 90 per cent of the cortical region after 24 hours ; few viable tumour cells were present 72 hours after irradiation.

### DISCUSSION

From the histological observations, it appeared that the viability of tumour cells irradiated with a single dose of 250 r was not affected. The effect of this dose on the growth rate of this tumour would therefore be a period of delay corresponding to the period of mitotic inhibition. This delay of about 8 hours is

---

EXPLANATION OF PLATE

Cortical region of Sarcoma 37. Fixed in Susa and stained with haematoxylin and eosin.

FIG. 3.—Control tumour fixed 24 hours after commencement of experiment. × 250.
FIG. 4.—24 hours after irradiation with 250 r. × 250.
FIG. 5.—24 hours after irradiation with 1000 r. × 250.
FIG. 6.—72 hours after irradiation with 1000 r. × 250.

3

4

5

6

barely detectable and the growth rate approaches that of the control tumours (Fig. 1). A summation effect, based on the period of mitotic delay, with four fractions of 250 r at 6-hour intervals would give a delay in tumour growth of 24 hours or less over the control growth rate. The delay in growth, however, is of the order of 6 to 7 days. It would appear that under these conditions the important factor is the recovery period after irradiation which affects the viability of the cell. A considerable proportion of the cortex of tumours irradiated at 12-hour intervals, and fixed 48 hours after the last irradiation, consisted of dead cells. This period of recovery was greater than 12 hours after irradiation, although some recovery took place at irradiation intervals of 15 minutes to 12 hours as the summation of these doses was not complete.

This recovery period might be allied to structural damage of the cell with subsequent repair—in which event the fractionation of a given dose of irradiation might not produce an effect greater than the total dose (super-summation). This concept agrees with previous data on fractionation (with the exception of Fogg and Cowing (1953)), where no effects greater than the single total dose were shown. If the recovery period was concerned with damage during some mitotic or intermitotic phase, converting the cell to a postmitotic condition, a suitable choice of time interval and dose might produce a super-summation effect.

### SUMMARY

1. The effects of fractionated doses of X-irradiation on the growth of Sarcoma 37 in RIII mice have been studied.

2. With time-intervals of 15 minutes, 1 hour, 3 hours, 6 hours, 12 hours and 24 hours, no increased inhibitory effect over the total dose was observed.

3. Fractionations at time-intervals of from 15 minutes to 12 hours produced comparable growth inhibition.

4. The effects of the fractionation treatments were not related solely to periods of mitotic delay.

5. The action of fractionation treatments in relation to a recovery period is discussed.

My thanks are due to Mr. F. W. Butcher and Mrs. C. E. Warren for assistance with the animal experiments.

The expenses of this research were defrayed from a block grant by the British Empire Cancer Campaign.

### REFERENCES

COHEN, L.—(1952) *Brit. J. Radiol.*, **25**, 636.
DU SAULT, L. A.—(1956) *Amer. J. Roentgenol.*, **75**, 597.
FOGG, L. C. AND COWING, R. F.—(1953) *Cancer Res.*, **13**, 321.
FRIEDMAN, N. B., SARGENT, J. A. AND DRUTZ, E.—(1955), *Ibid.*, **15**, 479.
HEEREN, I. E.—(1948) *Strahlentherapie*, **77**, 383.
LAMARQUE, P. AND GARY-BOBO, J.—(1955) *International Conference on Peaceful Uses of Atomic Energy*, **11**, 351.
QUIMBY, E. AND MACCOMB, W. S.—(1937) *Radiology*, **29**, 305.
REISNER, A.—(1932) *Fortschr. Röntgenstr.*, **45**, 293.
SCHÖTTELNDREYER, H.—(1949) *Brit. J. Radiol.*, **22**, 386.
WARREN, S. L. AND WHIPPLE, G. H.—(1923) *J. exp. Med.*, **38**, 725.